# Supplementary material for: Combining Ability and Inheritance Nature of Agronomic Traits and Resistance to Pink Stem (Sesamia cretica) and Purple-Lined (Chilo agamemnon) Borers in Maize
Source: Plants (Basel). 2023 Mar 1;12(5):1105. doi: 10.3390/plants12051105 (PMC10005691; doi:10.3390/plants12051105)
Supplement: Supplementary file 1 [file plants-12-01105-s001.zip › plants-2231920-supplementary.pdf]

Table S1. Code, name, pedigree and source of the seven maize inbred lines

Parent Code	Pedigree	Source
IL1	Rg-14 g.s (Syn. Laposta x Ci 64) (SC.14)	ARC-Egypt
IL2	G-504 B Improved by BC with (64 x 213)	ARC-Egypt
IL3	Rg-33 g.s (PI221866 x 307 A) (SC.14)	ARC-Egypt
IL4	G -268 Jellicarse (from R selection)	ARC-Egypt
IL5	[7480{TZVAR}/TZSR]-Y-1-345-1-1-1-1-S5-2-5-4-4-4-b-b	CIMMYT-Mexico
IL6	[7794]-SELF-4-1-S9-1-4-7-4-5-b-b	CIMMYT-Mexico
IL7	[EMSR]#B#bF101sr-2-1-sr-3-2-4-b-b	CIMMYT-Mexico

Table S2. Some physical and chemical soil characteristics of the experimental sites during 2020 and 2021 growing seasons

Properties	**2020**	**2021**
Soil texture (%)	Clay	Clay
Sand %	14.33	13.80
Silt %	34.27	33.90
Clay %	51.40	52.30
pH (1: 2.5 water suspension)	8.5	7.98
EC (dSm^{-1})	3.30	3.56
Organic matter	1.76	1.52
Available Nitrogen, mg kg-1	33.56	30.75
Available phosphorus. Mg kg-1	10.20	10.6
Available potassium, mg kg-1	280.54	260.6
